# Modulation of sleep behavior in zebrafish larvae by pharmacological targeting of the orexin receptor

**DOI:** 10.3389/fphar.2022.1012622

**Published:** 2022-10-10

**Authors:** Marie Pardon, Pieter Claes, Sarah Druwé, Murielle Martini, Aleksandra Siekierska, Christel Menet, Peter A. M. de Witte, Daniëlle Copmans

**Affiliations:** ^1^ Laboratory for Molecular Biodiscovery, Department of Pharmaceutical and Pharmacological Sciences, KU Leuven, Leuven, Belgium; ^2^ Confo Therapeutics, Ghent, Belgium

**Keywords:** zebrafish, orexin, sleep disorders, pharmacological analysis, functional analysis, and behavior

## Abstract

New pharmacological approaches that target orexin receptors (OXRs) are being developed to treat sleep disorders such as insomnia and narcolepsy, with fewer side effects than existing treatments. Orexins are neuropeptides that exert excitatory effects on postsynaptic neurons *via* the OXRs, and are important in regulating sleep/wake states. To date, there are three FDA-approved dual orexin receptor antagonists for the treatment of insomnia, and several small molecule oral OX2R (OXR type 2) agonists are in the pipeline for addressing the orexin deficiency in narcolepsy. To find new hypnotics and psychostimulants, rodents have been the model of choice, but they are costly and have substantially different sleep patterns to humans. As an alternative model, zebrafish larvae that like humans are diurnal and show peak daytime activity and rest at night offer several potential advantages including the ability for high throughput screening. To pharmacologically validate the use of a zebrafish model in the discovery of new compounds, we aimed in this study to evaluate the functionality of a set of known small molecule OX2R agonists and antagonists on human and zebrafish OXRs and to probe their effects on the behavior of zebrafish larvae. To this end, we developed an *in vitro* IP-One Homogeneous Time Resolved Fluorescence (HTRF) immunoassay, and *in vivo* locomotor assays that record the locomotor activity of zebrafish larvae under physiological light conditions as well as under dark-light triggers. We demonstrate that the functional IP-One test is a good predictor of biological activity *in vivo*. Moreover, the behavioral data show that a high-throughput assay that records the locomotor activity of zebrafish throughout the evening, night and morning is able to distinguish between OXR agonists and antagonists active on the zebrafish OXR. Conversely, a locomotor assay with alternating 30 min dark-light transitions throughout the day is not able to distinguish between the two sets of compounds, indicating the importance of circadian rhythm to their pharmacological activity. Overall, the results show that a functional IP-one test in combination with a behavioral assay using zebrafish is well-suited as a discovery platform to find novel compounds that target OXRs for the treatment of sleep disorders.

## 1 Introduction

Sleep is a complex process, regulated by circadian and homeostatic processes, and influenced by genetic and environmental factors (Ahmad et al., 2021). Sleep disorders, including apneas, insomnia, narcolepsy, parasomnias, and circadian rhythm disorders, are prevalent and can seriously affect patients’ quality of life (Ramar and Olson, 2013; Ahmad et al., 2021). Common treatment options for insomnia include hypnotics like benzodiazepines, while in the case of narcolepsy sedatives like sodium oxybate, that improve nighttime sleep or amphetamine-like psychostimulants that improve daytime wakefulness are typically used. However, these therapeutics are associated with major side effects and therefore new pharmacological avenues are being pursued to develop novel compounds that combine efficacy and safety (Nishimura et al., 2015).

A new approach to treat sleep disorders exploits the orexin receptors (Sun et al., 2021). Orexins (or hypocretins) are neuropeptides produced by orexin neurons in the lateral hypothalamus with excitatory effects on postsynaptic neurons *via* the orexin receptors (OXR) (Mahoney et al., 2019; Li and de Lecea, 2020). Orexin A (OXA) activates the orexin or hypocretin receptor type 1 (OX1R/HcrtR1) and type 2 (OX2R/HcrtR2) with roughly equal potency, whereas Orexin B (OXB) has about 10x higher potency for OX2R over OX1R (Barateau and Dauvilliers, 2019). Orexin neurons are distributed within the lateral hypothalamus and are also found throughout the brain and spinal cord (Mieda, 2017). The extensive range of their connections indicates that the orexin neurons act as integrators of a large variety of neuronal signals (Mahoney et al., 2019). Orexins are important in regulating sleep/wake states, likely by stimulating awake-active monoaminergic neurons. In addition, the orexin system also transmits to other brain functions, including energy homeostasis, reward processing, emotion and arousal (Mieda, 2017). Significantly, the loss of orexin-producing neurons has been associated with narcolepsy, a sleep disorder characterized by excessive daytime sleepiness and cataplexy. For example, narcolepsy phenotypes were observed in prepro-orexin knockout (KO) mice, in dogs with mutations in the gene encoding OX2R, and low levels of orexins were detected in the cerebrospinal fluid (CSF) of narcolepsy patients (Chemelli et al., 1999; Lin et al., 1999; Nishino et al., 2000; Adamantidis et al., 2020). It has been suggested that autoimmunity underlies the pathogenesis of this disease (Dye et al., 2018).

To date, there are three FDA-approved dual orexin receptor antagonists (DORAs) for the treatment of insomnia, i.e. suvorexant (Merck), lemborexant (Eisai) and daridorexant (Idorsia) (Bennett et al., 2014; Mezeiova et al., 2020; Kaushik et al., 2021). Conversely, agonizing the orexin receptors could be a treatment option for addressing the orexin deficiency in narcolepsy (Barker et al., 2020) and several small molecule oral OX2R agonists are currently in the pipeline (Bassetti et al., 2019; Zeitzer, 2021). Most notably TAK-925 (danavorexton) has shown promise in both preclinical and clinical studies (Sun et al., 2021).

Rodents are currently the model of choice to develop new hypnotics and psychostimulants. However, screening large numbers of compounds using mammalian models has several drawbacks, including high costs, low-throughput testing, and ethical considerations (Bruni et al., 2014; Nishimura et al., 2015). In addition, when preclinical models are used to study sleep disorders, typical animal-related sleep duration and patterns are of concern (Levitas-Djerbi and Appelbaum, 2017). Rodents are nocturnal, meaning that their sleep pattern is polyphasic and their main sleep phase is during the day, while in humans, the circadian distribution of sleep tends to be consolidated and monophasic, with the main sleep phase occurring at night (Zhdanova, 2006; Toth and Bhargava, 2013; Tisdale et al., 2021). In addition, humans go through 4-6 cycles of non-rapid eye movement (NREM) and REM sleep at night, while in rodents, NREM and REM sleep cycles are much shorter and occur periodically throughout the day. Besides, sleep in rodents is highly fragmented, even under normal conditions (Toth and Bhargava, 2013).

Given these limitations, the use of zebrafish larvae offers an interesting alternative (Nishimura et al., 2015; Elbaz et al., 2017). Zebrafish are small vertebrates that can be used for high-throughput screenings in the context of drug discovery and development, thereby reducing costs and the need for mammals (Nishimura et al., 2015; Miyawaki, 2020). They possess similar brain structures as other vertebrates including the neurochemical pathways necessary for generating and maintaining sleep (Zhdanova, 2006; Leung et al., 2019). For instance, the sleep behavior of zebrafish, which is characterized primarily by periods of reversible immobility with an increased arousal threshold, has been studied in several behavioral assays to assess differences in sleep-wake states after pharmacological or genetic interventions, and to study the mechanisms of sleep (Bruni et al., 2014; Nishimura et al., 2015; Leung et al., 2019). In addition, zebrafish, like humans, are diurnal and show peak daytime activity and rest at night (Elbaz et al., 2013; Nishimura et al., 2015). Finally, adult and larval zebrafish show sleep recovery in response to sleep deprivation and respond to different types of hypnotics (Zhdanova, 2006; Leung et al., 2019). As a result, zebrafish are gaining popularity as a non-mammalian vertebrate model to study sleep-related processes and sleep disorders (Zhdanova, 2006; Levitas-Djerbi and Appelbaum, 2017).

The orexin neuronal network has been studied in zebrafish, mainly because of its association with narcolepsy (Elbaz et al., 2013). The zebrafish orexin network contains only 16–60 neurons (about 100-times less compared to mammals), which are located in the hypothalamus, and extensively innervate the brain and spinal cord (Prober et al., 2006; Elbaz et al., 2017). As with mammals, zebrafish’s orexin network is involved in the regulation of several fundamental behaviors, such as feeding, sleep and wakefulness (Prober et al., 2006; Elbaz et al., 2017). For example, in the study by Prober and others, overexpression of orexin in zebrafish larvae increased arousal and decreased the ability to initiate and maintain a sleep-like state, similar to insomnia in humans (Prober et al., 2006). Similar to mammals, the zebrafish orexin gene consists of two exons, encoding two orexin neuropeptides (OXA/B) (Prober et al., 2006; Elbaz et al., 2017). Only one orexin receptor has been identified in zebrafish, which is more closely related to the mammalian OX2R (70%) than OX1R (60%). Interestingly, the binding pocket is highly conserved, with only three semiconserved mutations between the zebrafish and mammalian OX2R (Figure 8) (Prober et al., 2006; Isberg et al., 2015; Elbaz et al., 2017). Overall, the simplicity of the zebrafish orexin system makes this vertebrate an interesting model to investigate the different functions of the orexins’ neuronal network (Elbaz et al., 2017).

The present study aimed to evaluate functionality of small molecule OX2R agonists and antagonists on human and zebrafish OXRs and probe their effect on the behavior of zebrafish larvae. To this end, we have developed an *in vitro* IP-One HTRF (Homogeneous Time Resolved Fluorescence) immunoassay, and an *in vivo* locomotor activity assay that records the locomotor activity of zebrafish larvae during the evening, night and morning.

## 2 Materials and methods

### 2.1 Compounds

Suvorexant (Bio-Connect, Netherlands), TCS-1102 (Tocris Bioscience, United Kingdom), EMPA (Tocris Bioscience, United Kingdom), SB-674042 (Sigma-Aldrich, Germany), TAK-925 (Enamine Ltd, Ukraine), C15454 (WO 2019/027058, example 484, DSK InnoSciences, India), and C19069 (DSK InnoSciences, India) were obtained as dry powder and dissolved in 100% dimethyl sulfoxide (DMSO, spectroscopy grade, Acros Organics, Belgium) as 100-fold concentrated stocks (stored at −20°C). For zebrafish experiments, the compound stock solutions were diluted in embryo medium to a final concentration of 1% DMSO. Control groups were treated with vehicle (VHC, 1% DMSO in embryo medium).

### 2.2 Cell culture and transfection

CHO cells stably overexpressing full length human wild type-OX2R (Uniprot ID O43614) carrying an N-terminal Flag tag were maintained in DMEM/F12 with 5% heat inactivated fetal bovine serum, 0.8 mM l-glutamine and 0.5 mg/ml Geneticin. F293 suspension cells were transiently transfected with a full-length zebrafish OX2R (Uniprot ID A7KBS6) construct carrying an N-terminal Flag tag in pCDNA3.1(+) or a full length human OX2R construct carrying an N-terminal Flag tag and mutations T111S, I130L and V353I in pCDNA3.1 (+).

F293 suspension cells were maintained in Freestyle medium. Cells were seeded 24 h before transfection at a density of 0.6 million cells/ml. The day of the transfection, 1 µg DNA per million cells was transfected using X-tremeGene HP (Roche, Switzerland) according to the manufacturer’s instructions. Cells were harvested after 24 h (at 37°C, relative humidity of 95% and 5% CO_2_), frozen (10 million cells/ml) and stored at −80°C.

### 2.3 IP-one HTRF assay

IP-One assays were conducted by using a homogeneous time-resolved fluorescence resonance energy transfer (TR-FRET) immunoassay (Perkin Elmer, IP-One Gq kit, cat #62IPAPEC) according to the manufacturer’s instructions. The kit is based on a competitive format involving a specific antibody labeled with cryptate (donor) and IP1 coupled to d2 (acceptor).

The assay was performed in 24 µl final volume. Frozen cells were thawed and washed in HBSS (W/O Mg and Ca; Cat# 14175095, Gibco) with 20 mM HEPES (Cat# 15630056, Gibco) at pH 7.4. After a centrifugation (5 min at 200 g, RT), cells were resuspended in the assay buffer (Stimulation buffer 1x from the kit) containing LiCl (causing IP1 accumulation upon receptor activation). Cells were seeded in 96 well half area white plates (Cat# 675075, Greiner), between 20 000–25 000 cells/well (depending on the expression level). Plates were covered and stabilized for 45 min at 37°C. In agonist mode, 6 µl of assay buffer was added, the plates were covered and incubated for 30 min at 37°C. Then 6 µl of compound 4x concentrated in stimulation buffer 1X was added, the plates were covered and incubated for 60 min at 37°C. In antagonist mode, 6 µl of compound 4x concentrated in stimulation buffer 1X was added, the plates were covered and incubated for 30 min at 37°C. Then, 6 µl of reference agonist C14917 (WO 2016/199906, example 133) 4x concentrated in stimulation buffer 1X was added, the plates were covered and incubated for 120 min at 37°C.

The d2-conjugate and anti-IP-One cryptate conjugate diluted in lysis buffer (Perkin Elmer, IP-One Gq kit) were added sequentially into each well according to the manufacturer’s instructions. After 60 min incubation at room temperature in the dark, time-resolved FRET signals were measured at 620 nm and 650 nm by an EnVision 2104 Multilabel Plate Reader (Perkin Elmer). Data were analyzed using GraphPad Prism 9 (San Diego, CA, United States). Antagonist inhibition constants (K_i_) were calculated from the corresponding IC_50_ values using the Cheng-Prusoff equation (Lazareno and Birdsall, 1993):

$$K_{i}=\frac{{IC}_{50}}{1+\frac{\left[S\right]}{{EC}_{50}}}$$

Where IC_50_ is the molar concentration of antagonist that corresponds to 50% inhibition of the maximal signal, [S] is the concentration of agonist used in the assay and EC_50_ is the molar concentration of reference agonist that corresponds the 50% of the maximal signal (as determined in the same assay).

### 2.4 Zebrafish husbandry

Wild-type (WT) zebrafish (*Danio rerio*) of the AB strain were maintained at 28°C on a 14/10 h light/dark cycle under standard aquaculture conditions in a UV-sterilized rack recirculating system equipped with a mechanical and biological filtration unit. Embryos were collected *via* natural spawning and immediately transferred to a Petri dish containing Danieau’s medium (0.3x Danieau’s medium), which consists of a 1.5 mM HEPES buffer at pH 7.2, 17.4 mM NaCl, 0.21 mM KCl, 0.18 mM Ca(NO_3_)_2_, 0.12 mM MgSO_4_, and 0.6 mM methylene blue. Embryos and larvae were kept at 28.5°C on a 14/10 h light/dark cycle. All zebrafish experiments were performed in accordance with the guidelines of and approval by the Ethical Committee of the KU Leuven (P027/2019) and by the Belgian Federal Department of Public Health, Food Safety and Environment (LA1210261).

### 2.5 Toxicity evaluation

The maximum tolerated concentration (MTC) of compounds was determined as described previously (Copmans et al., 2018; Copmans et al., 2019) and used as the highest possible test concentration for the behavioral assays (i.e., night assay and dark-light assay). In brief, 12 larvae of 6 dpf were individually exposed to a certain concentration of test compound within a concentration range (2-fold dilution series, ranging between 500—3 µM) in a 100 µl volume in a 96-well plate at 28°C in the dark. After 24 h, overall morphology, heartbeat, and touch response were investigated by visual evaluation of zebrafish larvae under a light microscope. The MTC was defined as the highest concentration at which no larvae died nor showed signs of toxicity or locomotor impairment in comparison to VHC-treated control larvae. In case no MTC was reached, the highest soluble concentration was used as the highest test concentration.

### 2.6 Behavioral assays

**FIGURE 1:**
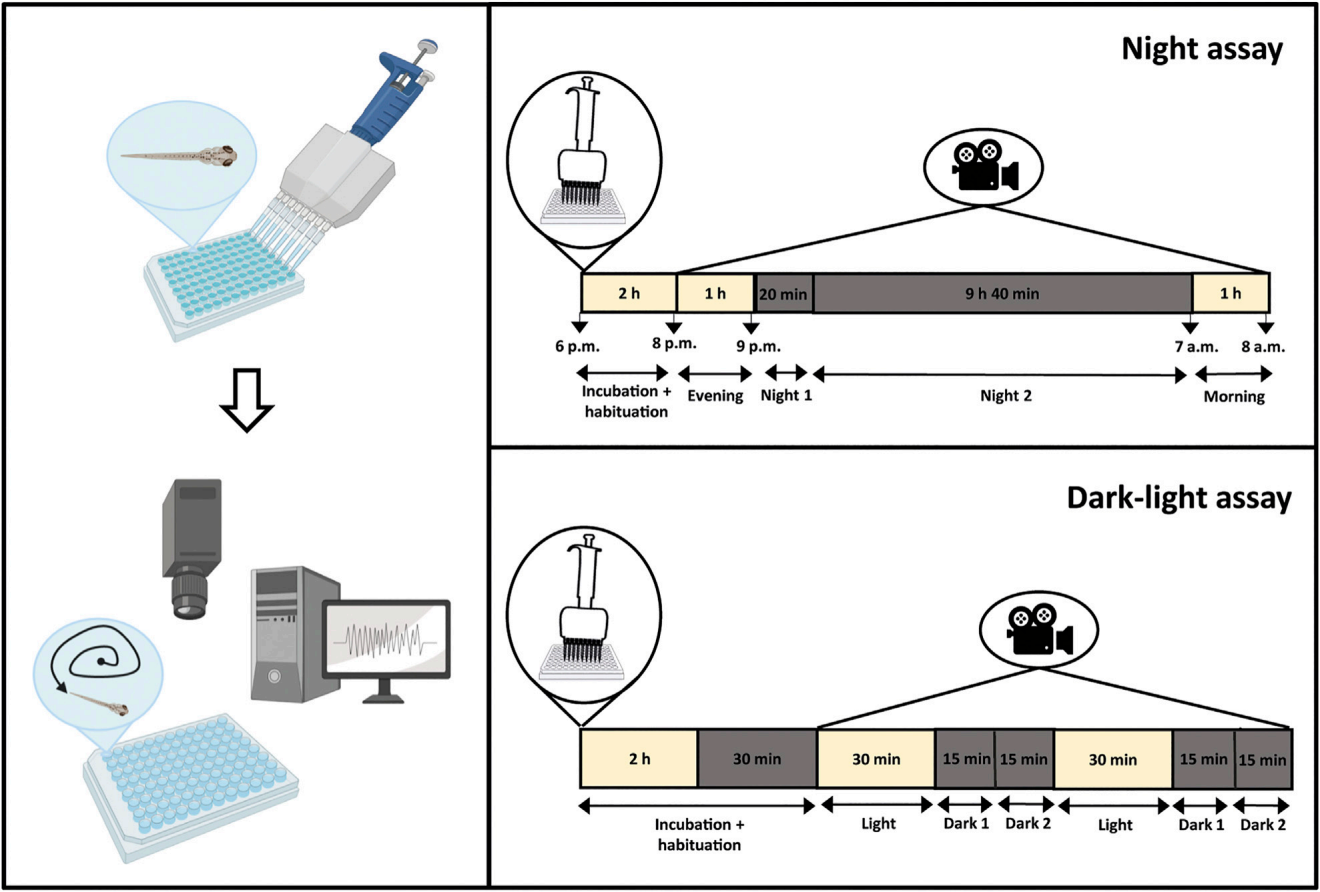
Behavioral analysis of zebrafish larvae. Zebrafish larvae were placed separately in a 96-well plate and incubated with compound or vehicle (VHC, 1% DMSO in embryo medium). After incubation, the 96-well plate was placed in an automated tracking device and larval behavior was recorded for varying times by the Zebralab software. The night assay consisted of a 2 h incubation and habituation period, followed by a 12 h recording period with a 1 h light phase from 8 p.m to 9 p.m., a 10 h dark phase from 9 p.m to 7 a.m., and again a 1 h light phase from 7 a.m to 8 a.m. The dark-light assay consisted of a 2 h 30 min incubation and habituation period, followed by a 2 h recording period with alternating 30 min dark-light triggers during the day. Figure created with BioRender.com.

A night assay and a dark-light assay were used to examine the locomotor behavior of 6 dpf zebrafish larvae (Figure 1). Locomotor activity was expressed in actinteg units per min for the dark-light assay, and per 10 min for the night assay, respectively, which represents the sum of pixel changes during the defined time interval. Data of three independent experiments were pooled together and expressed as mean actinteg (±SD) per light/dark phase, as specified below, and over specific time intervals (mean only) during the entire recording period. Data were analyzed using GraphPad Prism 9 (San Diego, CA, United States).

#### 2.6.1 Night assay

Zebrafish larvae were individually placed in single wells of a 96-well plate, and compound or VHC (1% DMSO in embryo medium) was added. To prevent evaporation of the embryo medium during this long-term assay, the wells were completely filled and covered by a glass plate. The 96-well plates were placed in an automated tracking device (Zebrabox, Viewpoint, France) without prior incubation. Larval behavior was recorded by Zebralab software (Viewpoint, France) for a 14 h period with a light phase from 5–6 p.m to 9 p.m., a dark phase from 9 p.m to 7 a.m., and again a light phase from 7 a.m to 8 a.m. The first 2–3 h of this assay were considered as a habituation period and were excluded from the behavioral data analysis. Hence, the last hour of light from 8 p.m. on, the first 20 min and the following 9 h 40 min in dark conditions, and the final 1 h light between 7 a.m and 8 a.m. were used and referred to as “Evening”, “Night 1”, “Night 2” and “Morning”, respectively (Figure 1).

#### 2.6.2 Dark-light assay

In the dark-light assay, zebrafish larvae were individually placed in single wells of a 96-well plate and incubated in the light for 2 h with compound or VHC (1% DMSO in embryo medium) in a 100 µl volume. This assay was carried out during the day, between 9 a.m and 6 p.m. After incubation, 96-well plates were placed in an automated tracking device (Zebrabox, Viewpoint, France), and larval behavior was recorded by Zebralab software (Viewpoint, France) for 2 h 30 min with alternating dark-light periods of 30 min. The first 30 min in the dark were considered as habituation and were excluded from the behavioral data analysis. Conversely, the next light phase of 30 min, followed by the first and last 15 min of the dark phase were used and referred to as “Light”, “Dark 1” and “Dark 2”, respectively (Figure 1).

#### 2.6.3 Statistical analysis

Statistical analyses of behavioral data were performed using two-way ANOVA (more than two groups and two variables) with Dunnett’s multiple comparison test in GraphPad Prism 9 (San Diego, CA, United States). Data are expressed as mean ± SD. Animals were randomly allocated to experimental groups.

## 3 Results

### 3.1 Functionality of orexin (ant)agonists on zebrafish OX2R

**FIGURE 2:**
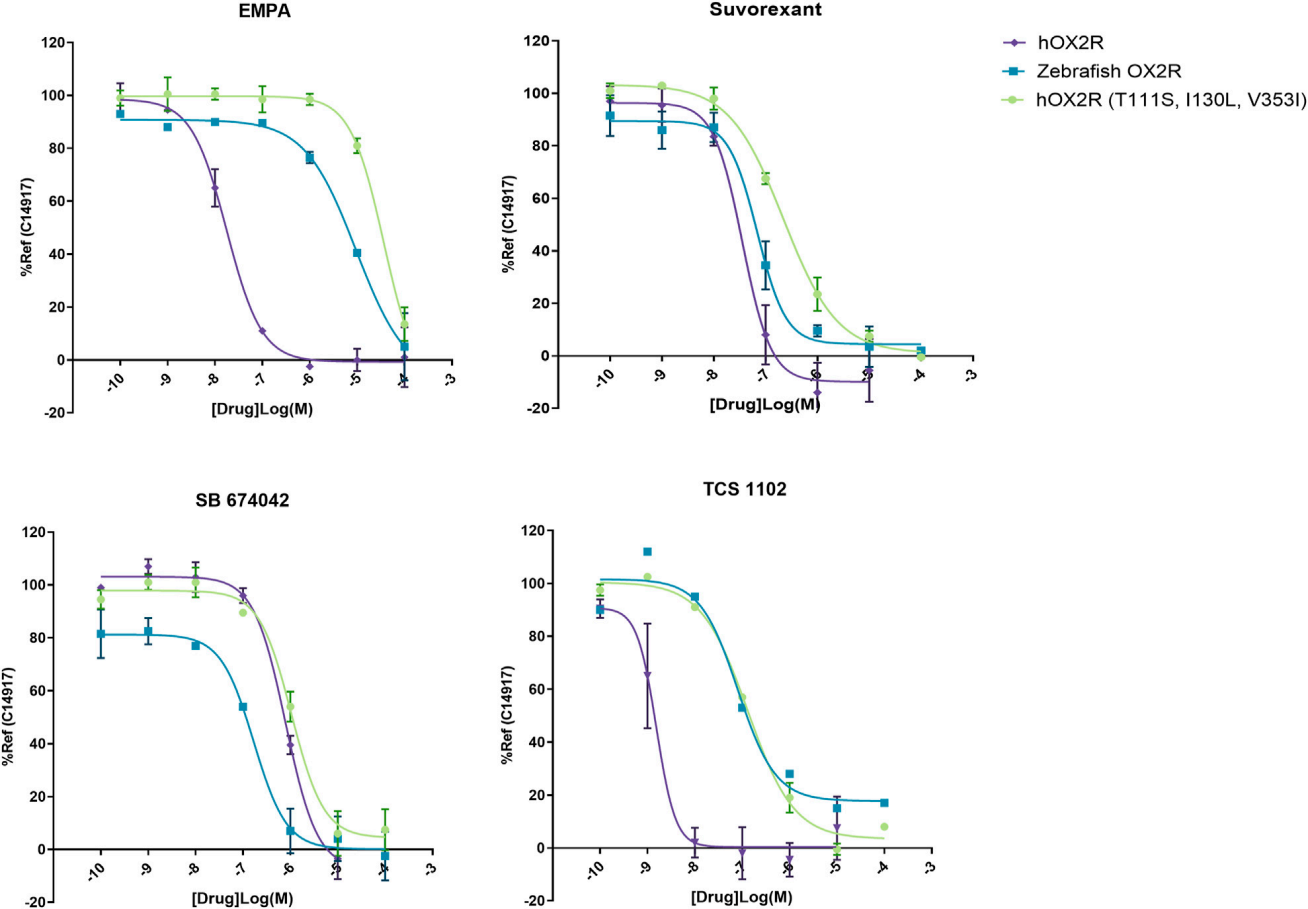
Representative IP-One dose response curves for antagonists EMPA, Suvorexant, SB 674042 and TCS 1102 on human OX2R (purple), zebrafish OX2R (blue) and hOX2R (T111S, I130L, V353I) (green). Antagonists were incubated for 30 min at 37°C and subsequently the cells were stimulated with 1 µM of reference agonist C14917. Cells were incubated for 2 more hours and subsequently readout was performed. Dose responses were performed as technical duplicates, data were normalized to 10 µM of reference agonist C14917.

DORA’s Suvorexant and TCS 1102, selective hOX2R antagonist (SORA-2) EMPA and selective hOX1R antagonist (SORA-1) SB 674042 were selected to evaluate their functionality on zebrafish OXR. Their ability to block agonist-induced inositol monophosphate (IP1) accumulation was determined in the IP-One G_q_ assay on zebrafish OX2R, human OX2R and human OX2R bearing the three semi-conserved binding site mutations between the human and zebrafish receptor ((T111S, I130L, V353I)). A representative experiment is shown in Figure 2. The resulting IC_50_ values were converted to K_i_ values to obtain agonist independent data. Results of all experiments are summarized in Table 1.

**TABLE 1:** Evaluation of antagonists and agonists on human OX2R, zebrafish OX2R and human OX2R bearing zebrafish pocket mutations in an IP-One functional assay. Antagonist potency is represented as the negative log of the molar antagonist concentration for which 50% of receptors will be occupied (pKi), agonist potency as the negative log of the molar agonist concentration for which 50% of the maximum stimulation is observed (pEC50). Data are averages of at least 3 independent repeats with the exception of EMPA and C19069.

Pharmacology	Ligand	IP-one pK_i_ (antagonist) or pEC_50_ (agonist)		
		hOX2R	Zebrafish OX2R	hOX2R (T111S, I130L, V353I)
DORA	Suvorexant	8.32 ± 0.32 (n = 7)	8.23 ± 0.24 (n = 6)	8.20 ± 0.19 (n = 4)
DORA	TCS 1102	9.45 ± 0.52 (n = 5)	7.63 ± 0.46 (n = 6)	8.13 ± 0.30 (n = 4)
SORA-2	EMPA	8.48 ± 0.07 (n = 4)	6.13 ± 0.16 (n = 3)	5.78 ± 0.28 (n = 4)
SORA-1	SB 674042	6.89 ± 0.12 (n = 4)	7.71 ± 0.15 (n = 5)	7.41 ± 0.29 (n = 4)
hOX2R agonist	TAK-925	7.77 ± 0.28 (n = 9)	6.12 ± 0.41 (n = 7)	6.33 ± 0.24 (n = 4)
hOX2R agonist	C15454	7.78 ± 0.54 (n = 9)	7.52 ± 0.46 (n = 9)	7.80 ± 0.61 (n = 5)
hOX2R agonist	C19069	7.01 ± 0.23 (n = 5)	7.20 ± 0.15 (n = 4)	6.46 ± 0.19 (n = 3)

While a decrease in pK_i_ was observed for the hOX2R selective antagonist TCS 1102, the potency was maintained for dual antagonist suvorexant and hOX1R selective antagonist SB 674042. A dramatic drop in potency was observed for SORA-2 EMPA and this compound was excluded from further experiments (Table 1). All these changes are largely recapitulated by the pK_i_ data on the human receptor harboring the zebrafish pocket mutations, indicating that the differences observed stem from altered orthosteric binding.

**FIGURE 3:**
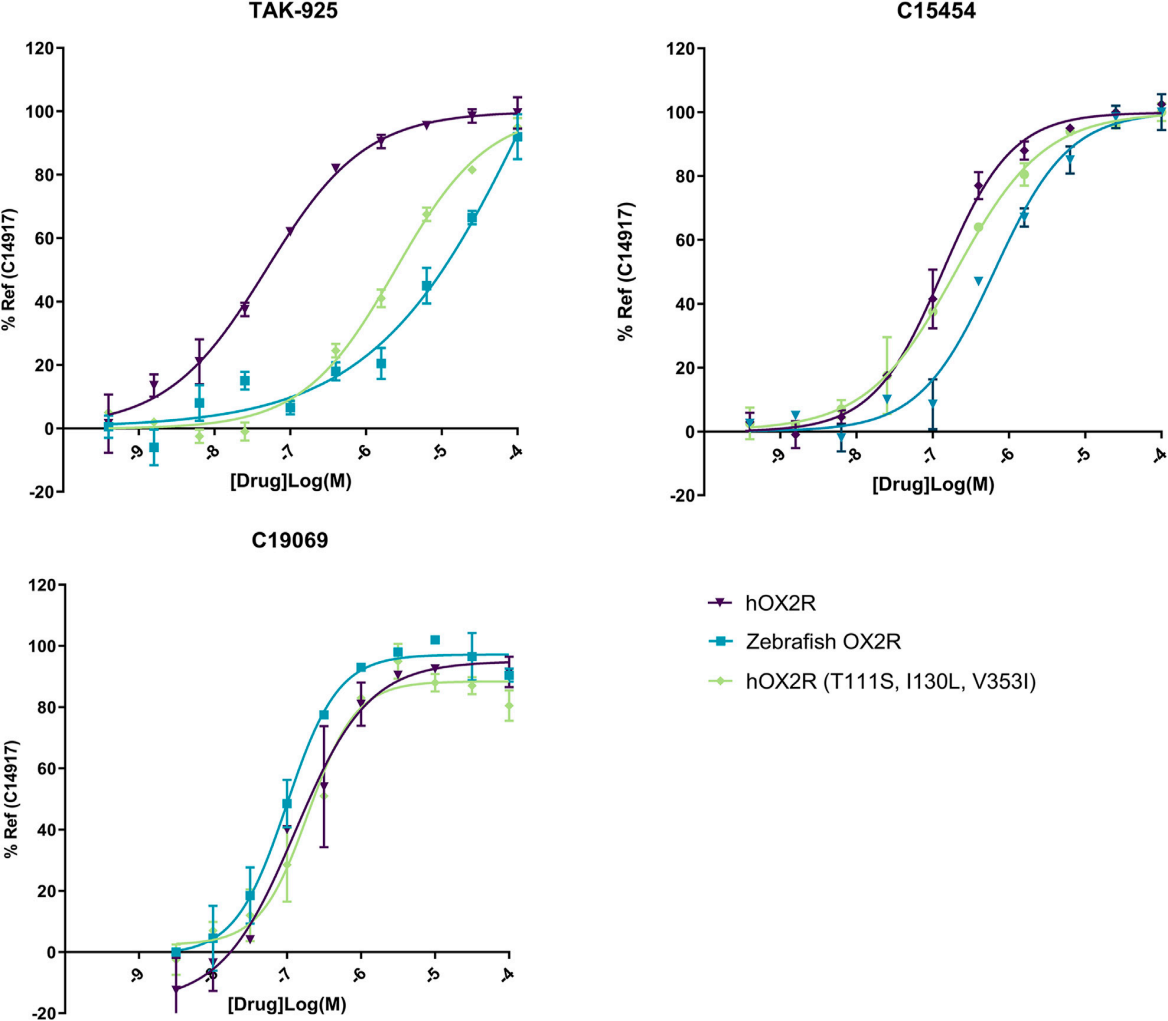
Representative IP-One dose response curves for agonists TAK-925, C15454 and C19069 on human OX2R (purple), zebrafish OX2R (blue) and hOX2R (T111S, I130L, V353I) (green). Cells were incubated for 1 h with agonist and subsequently readout was performed. Dose responses were performed as technical duplicates, data were normalized to 10 µM of reference agonist C14917.

To evaluate agonist pharmacology of the different receptors, TAK-925 (danavorexton) was selected as it is a potent OX2R selective agonist with demonstrated efficacy *in vivo* in both human and mice (Yukitake et al., 2019). However, a decrease in potency of nearly two logs on zebrafish OX2R relative to the human receptor was observed in IP-One functional test (Figure 3; Table 1). This decrease was maintained when testing the same compound on the human receptor containing the three zebrafish pocket mutations, indicating that the reduction is related to the pocket differences between human and zebrafish and not to any other aspect of the zebrafish receptor (Table 1). Conversely, C15454 as a potent, structurally distinct analogue of TAK-925, and the related compound C19069 both retained their potency on the zebrafish receptor (Table 1).

### 3.2 Influence of orexin receptor (ant)agonists on locomotor behavior of zebrafish larvae in the night assay

**FIGURE 4:**
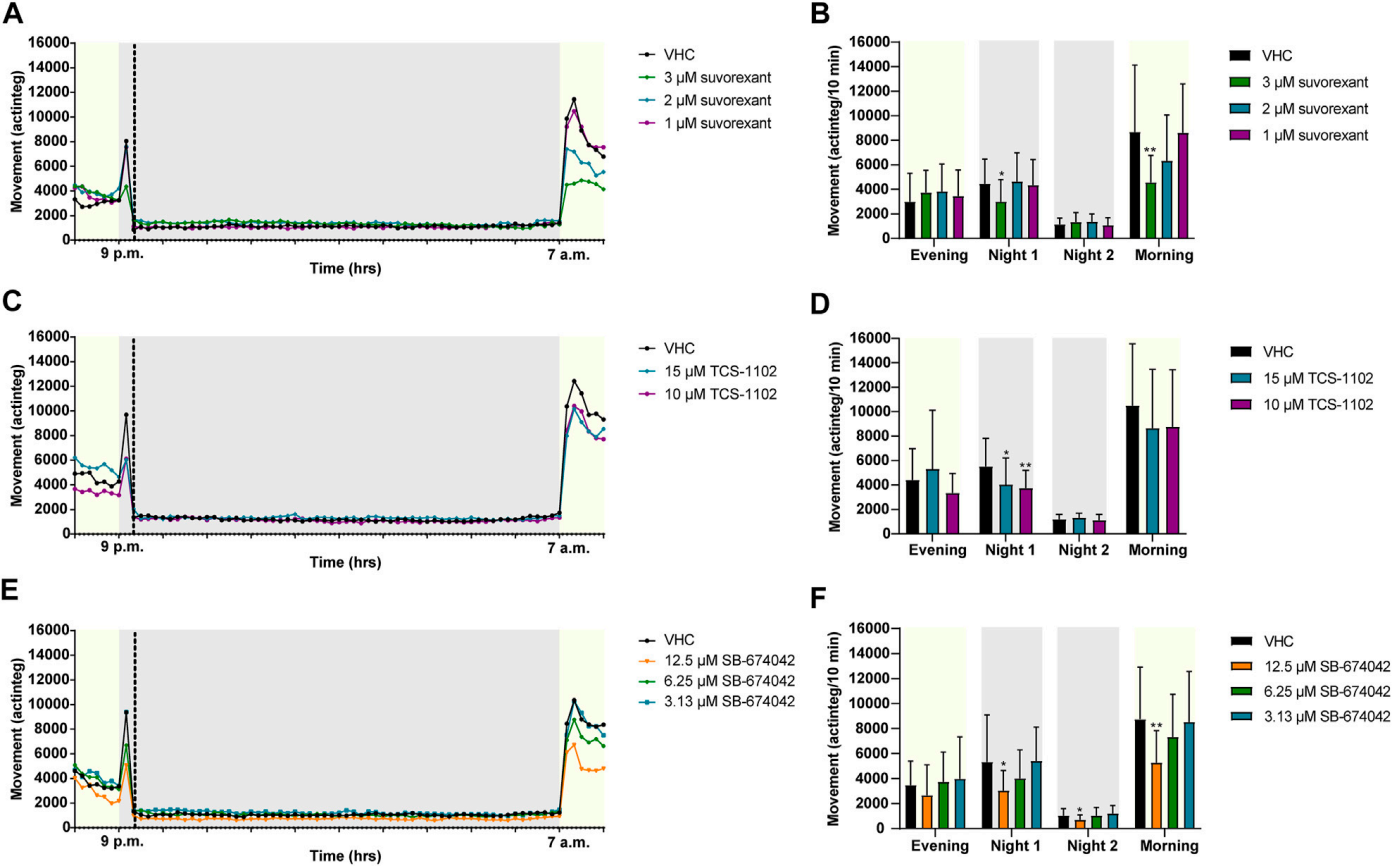
Behavioral analysis of 6 dpf zebrafish larvae treated with orexin receptor antagonists in the night assay. Locomotor profiles of animals are expressed as mean actinteg units per 10 min treated with suvorexant **(A)**, TCS-1102 **(C)** and SB-674042 **(E)** are plotted as function of time. Different phases are shown, i.e. the light phase from 8 p.m. to 9 p.m. (i.e., Evening), the first dark phase from 9 p.m. to 9:20 p.m. (i.e., Dark 1), the second dark phase from 9:20 p.m. to 7 a.m. (i.e., Dark 2), and the light phase from 7 a.m. to 8 a.m. (i.e., Morning). For the sake of clarity, standard deviations are not shown. Total locomotor activity was averaged (±SD) per respective light or dark phase **(B–F)** For each compound, data were pooled from three independent experiments with 9–10 replicate wells per test condition (*n* = 27–30, except for 15 µM TCS-1102 (*n* = 20)). Statistical analysis: two-way ANOVA with Dunnett’s multiple comparison test. Significance levels: *p ≤ 0.05, **p ≤ 0.01.

**FIGURE 5:**
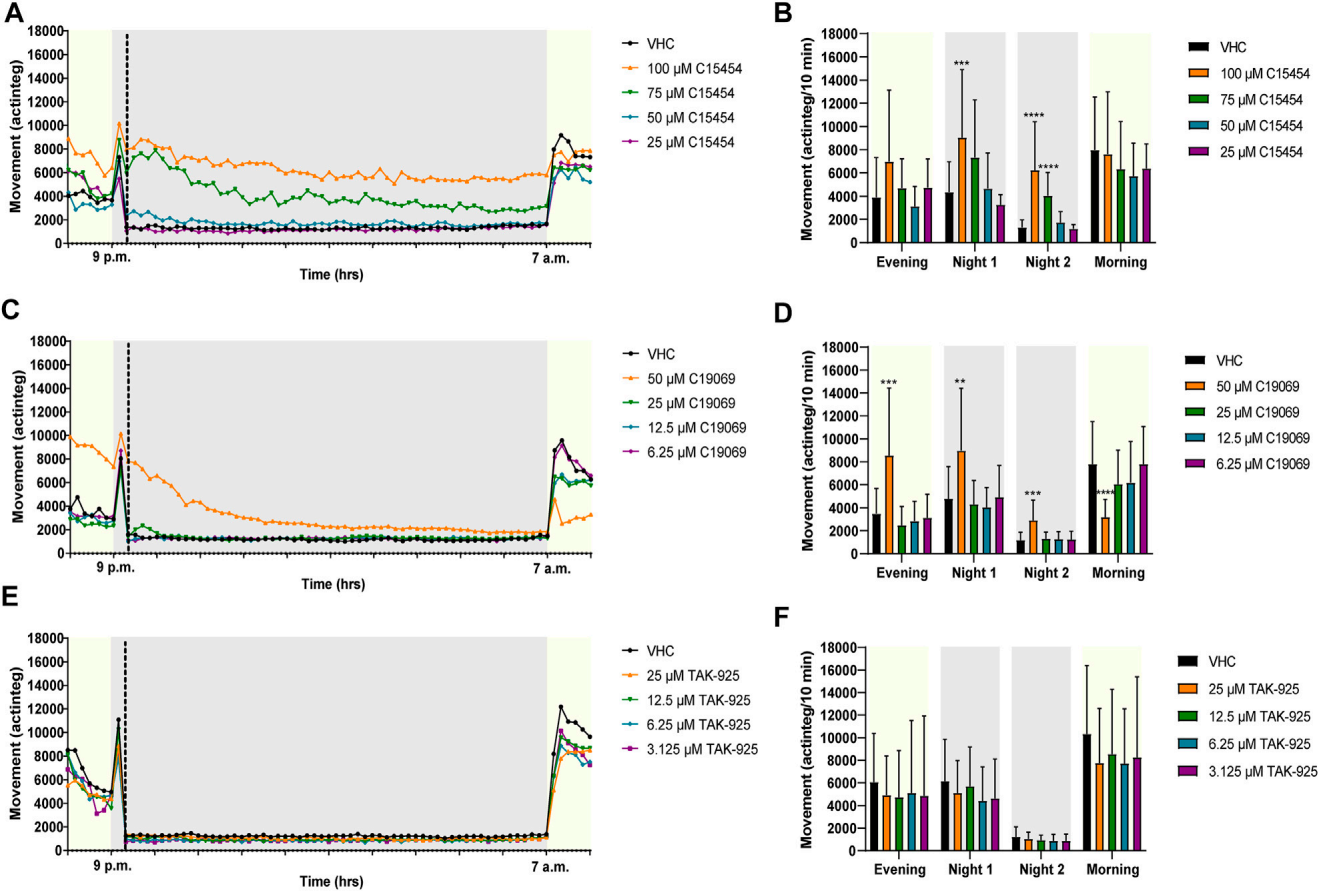
Behavioral analysis of 6 dpf zebrafish larvae treated with orexin 2 receptor agonists in the night assay. Locomotor profiles of animals are expressed as mean actinteg units per 10 min treated with C15454 **(A)**, C19069 **(C)** and TAK-925 **(E)** are plotted as function of time. Different phases are shown, i.e. the light phase from 8 p.m. to 9 p.m. (i.e., Evening), the first dark phase from 9 p.m. to 9:20 p.m. (i.e., Dark 1), the second dark phase from 9:20 p.m. to 7 a.m. (i.e., Dark 2), and the light phase from 7 a.m. to 8 a.m. (i.e., Morning). For the sake of clarity, standard deviations are not shown. Total locomotor activity was averaged (±SD) per respective light or dark phase **(B–F)**. For each compound, data were pooled from three or four independent experiments with 7–10 replicate wells per test condition (*n* = 23–30, except for 75 µM C15454 (*n* = 20) and 25 µM C15454 (*n* = 10)). Statistical analysis: two-way ANOVA with Dunnett’s multiple comparison test. Significance levels: **p ≤ 0.01, ***p ≤ 0.001, ****p ≤ 0.0001.

The effects of OXR (ant)agonists on the behavior of zebrafish larvae was investigated by locomotor activity analysis during a night assay, recorded between 6 p.m. and 8 a.m. (Figures 4, 5). Prior to behavioral assessment, the MTC of OXR antagonists and agonists was determined (Supplementary Table S1).

Larvae treated with OX2R antagonists suvorexant at 3 µM (*p* ≤ 0.05), TCS-1102 at 15 µM (*p* ≤ 0.05) and 10 µM (*p* ≤ 0.01), and SB-674042 at 12.5 µM (*p* ≤ 0.05) showed a significant reduction in locomotor activity during Night 1 phase in comparison to VHC-treated larvae (Figures 4B–F). In addition, SB-674042 at 12.5 µM significantly lowered the activity of zebrafish larvae compared to VHC-treated larvae during Night 2 phase (*p* ≤ 0.05) (Figure 4F), and larvae treated with suvorexant at 3 µM and SB-674042 at 12.5 µM showed a significantly lower activity in the Morning phase (*p* ≤ 0.01) (Figures 4B,F).

Larvae treated with OX2R agonist C19069, significantly (*p* ≤ 0.001) increased locomotor activity of zebrafish larvae at 50 µM during the Evening phase, compared to VHC-treated larvae (Figure 5D). During the first 20 min of the night (Night 1), agonists C15454 at 100 µM and C19069 at 50 µM significantly increased locomotor activity of zebrafish larvae compared to VHC-treated larvae (*p* ≤ 0.001, and *p* ≤ 0.01, respectively) (Figures 5B,D). During the Night 2 phase, C15454 at 100 µM and 75 μM, and C19069 at 50 µM significantly increased locomotor activity of zebrafish larvae compared to VHC-treated larvae (*p* ≤ 0.0001, and *p* ≤ 0.001, respectively) (Figures 5B,D). Interestingly, C19069 at 50 µM also significantly (*p* ≤ 0.0001) lowered the locomotor activity of zebrafish larvae in the Morning phase (Figure 5D). For TAK-925, no significant differences in zebrafish locomotor activity in comparison to VHC-treated larvae were observed during the entire night assay (Figure 5F).

### 3.3 Influence of orexin receptor (ant)agonists on locomotor behavior of zebrafish larvae in the dark-light assay

**FIGURE 6:**
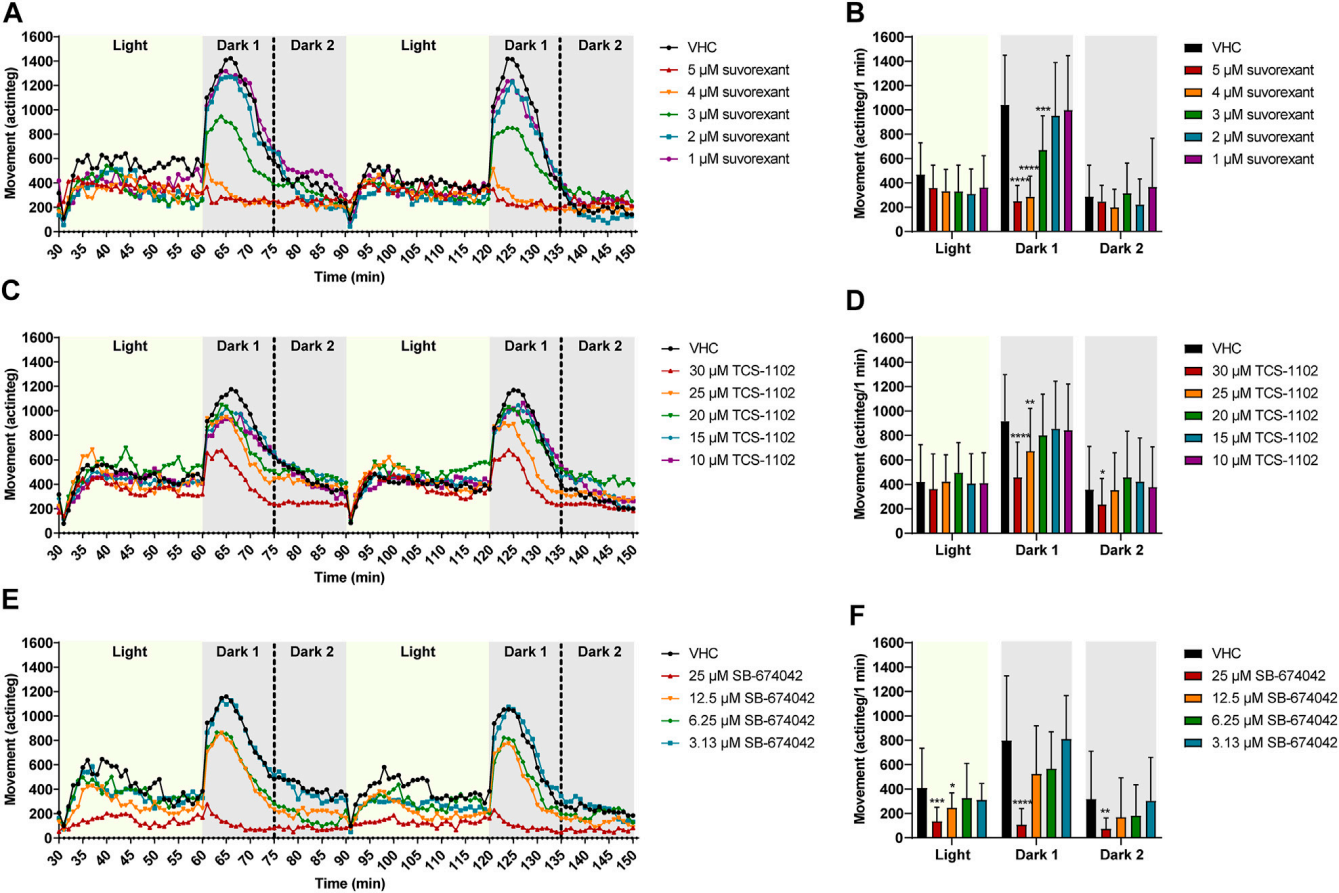
Behavioral analysis of 6 dpf zebrafish larvae treated with orexin receptor antagonists in the dark-light assay. Locomotor profiles of animals are expressed as mean actinteg units per 1 min treated with suvorexant **(A)**, TCS-1102 **(C)** and SB-674042 **(E)** are plotted as function of time. For the sake of clarity, standard deviations are not shown. Different phases are shown, i.e., 30 min light phase (Light), the first 15 min of the dark phase (Dark 1) and the last 15 min of the dark phase (Dark 2). Total locomotor activity was averaged (±SD) per respective light or dark phase **(B–F)**. For each compound, data were pooled from three or nine independent experiments with 9–20 replicate wells per test condition (*n* = 29–40, except for VHC **(C,D)** *n* = 110) and 30 µM TCS-1102 (*n* = 50)). Statistical analysis: two-way ANOVA with Dunnett’s multiple comparison test. Significance levels: *p ≤ 0.05, **p ≤ 0.01, ***p ≤ 0.001, ****p ≤ 0.0001.

**FIGURE 7:**
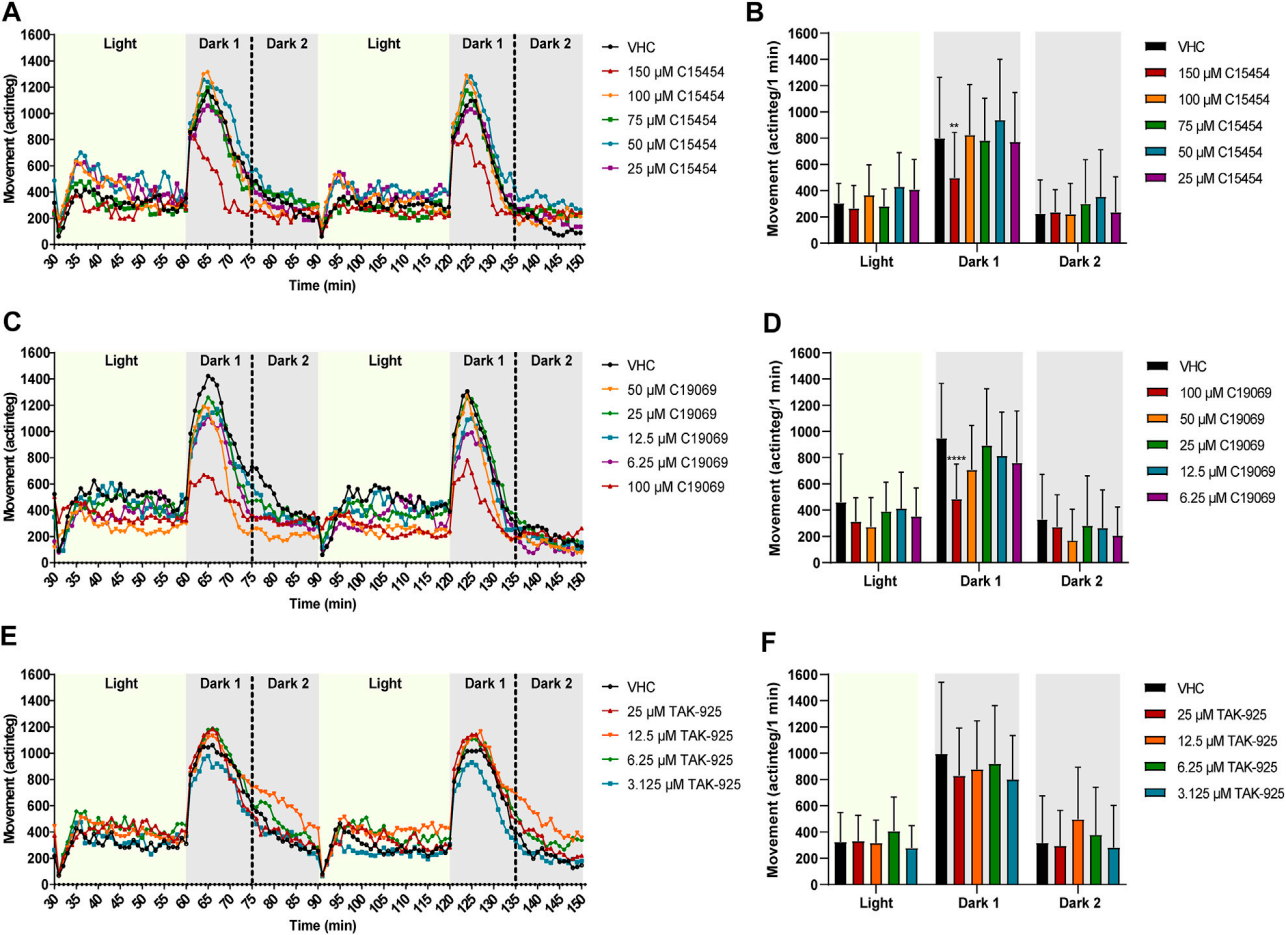
Behavioral analysis of 6-dpf zebrafish larvae treated with orexin 2 receptor agonists in the dark-light assay. Locomotor profiles of animals are expressed as mean actinteg units per 1 min treated with C15454 **(A)**, C19069 **(C)** and TAK-925 **(E)** are plotted as function of time. For the sake of clarity, standard deviations are not shown. Different phases are shown, i.e. 30 min light phase (Light), the first 15 min of the dark phase (Dark 1) and the last 15 min of the dark phase (Dark 2). Total locomotor activity was averaged (±SD) per respective light or dark phase **(B–F)**. For each compound, data were pooled from three or four independent experiments with 9–20 replicate wells per test condition (n = 29–30, except for VHC **(A,B)** *n* = 50) and 6.25—12.5 µM C19069 (*n* = 20)). Statistical analysis: two-way ANOVA with Dunnett’s multiple comparison test. Significance levels: **p ≤ 0.01, ****p ≤ 0.0001.

To investigate whether the effects observed in the night test were critically depending on the circadian rhythm and behavior of zebrafish larvae, OX2R (ant)agonists were tested in a dark-light test that consisted of a 2 h period with 30 min alternating dark-light phases performed throughout the day (Figures 6, 7).

In the dark-light assay, the antagonist suvorexant significantly lowered the behavioral response of zebrafish larvae during the Dark 1 phase at 5, 4, and 3 µM (*p* ≤ 0.0001, *p* ≤ 0.0001, and *p* ≤ 0.001, respectively) in comparison to VHC-treated larvae (Figure 6B). A clear concentration-dependent relationship was observed. TCS-1102 also significantly reduced the behavioral response after the light to dark transition at 30 and 25 µM (*p* ≤ 0.0001 and *p* ≤ 0.01, respectively) and SB-674042 at 25 µM (*p* ≤ 0.0001) (Figures 6D–F). During the last 15 min of the dark phases (i.e., Dark 2), TCS-1102 at 30 µM and SB-674042 at 25 µM significantly lowered the locomotor activity in comparison to VHC-treated larvae (*p* ≤ 0.05 and *p* ≤ 0.01, respectively) (Figures 6D–F). SB-674042 also significantly lowered the locomotor activity of zebrafish larvae during the light phases at 25 μM and 12.5 µM (*p* ≤ 0.001 and *p* ≤ 0.05, respectively) (Figure 6F).

Surprisingly, the OX2R agonists C15454 at 150 µM and C19069 at 100 µM significantly lowered the behavioral response of zebrafish larvae during the first 15 min of the dark phase (i.e., Dark 1) in comparison to VHC-treated larvae (*p* ≤ 0.01, and *p* ≤ 0.0001, respectively) (Figures 7B–D). For TAK-925, again no significant differences in locomotor activity of zebrafish were observed compared to the treated VHC larvae (Figure 7F).

## 4 Discussion

Since targeting of the orexin system is promising for the discovery and development of new and improved sleep and wake promoting agents, this study focused on comparing the functionality of small molecule OX2R agonists and antagonists on zebrafish OXRs *in vitro* and to investigate their effects *in vivo* on the behavior of zebrafish larvae.

**FIGURE 8:**
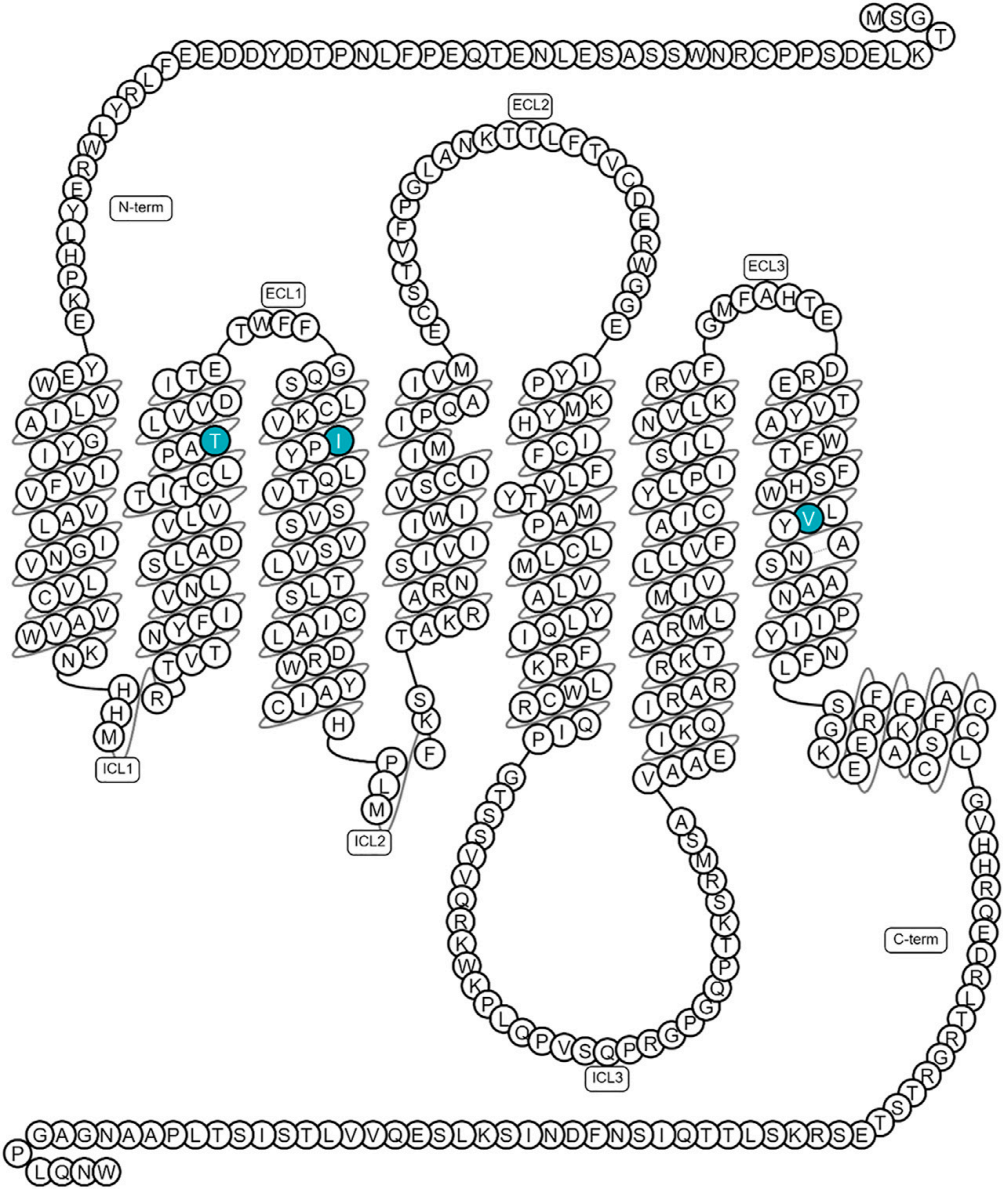
Location of the three semi-conserved binding site mutations mapped on human OX2R. Figure generated using GPCRdb (https://gpcrdb.org/).

Recently, both agonists and antagonists of OX2R have been shown to bind to the same orthosteric binding site at the structural level (Hong et al., 2021). At the binding site, there are three semi-conserved mutations between the human and zebrafish receptor: T/S^2.61x60^, I/L^3.28x28^ and V/I^7.42x41^ (Figure 8) with superscript numbers referring to the generic GPCRdb residue numbering scheme (Isberg et al., 2015). Because these mutations could affect ligand affinity, the different OXR antagonists and agonists were tested in an IP-One assay to assess their functionality in G_q_ mediated IP1 release through the zebrafish OXR.

Of the antagonist panel tested, suvorexant (Cox et al., 2010) was the most potent with a fully conserved functionality on the human and zebrafish OX2R. For SORA-1 SB 674042 a slight increase in pK_i_ was observed while a larger drop in functionality was observed for DORA TCS 102 when comparing the human and zebrafish receptor.

While multiple orexin antagonists are reported and commercially available, identification of small molecule agonists for the orexin receptors remains challenging, limiting the choice of appropriate tool compounds. For instance, the endogenous peptides orexin A and B cannot be used, as they cannot cross the blood brain barrier, and the tool compounds are to be administered by water immersion to utilize the high-throughput capacity of zebrafish larvae. Similarly, compounds like YNT-185 and Nag-26, the first reported non-peptide OX2R agonists are not suitable given their low passive permeability and limited solubility (Irukayama-Tomobe et al., 2017). Therefore, TAK-925 (Danavorexton) was selected as it is not only a potent and selective OX2R agonist, it also has demonstrated *in vivo* efficacy in both preclinical species and human in clinical studies (Yukitake et al., 2019). However, when testing TAK-925 in the Gq signaling assay (IP-one) on zebrafish OXR, a decrease in the potency of nearly two logs was found when comparing to the results obtained with testing on the human receptor. This could be explained at least in part by the presence of three zebrafish pocket mutations, as testing in the human receptor with the three zebrafish pocket mutations also showed a strong decrease in potency. C15454, a structurally distinct analogue of TAK-925 with reported *in vivo* activity in preclinical species, was then selected as a potent agonist of both human and zebrafish OXR. In table 1, C15454 and close analogue C19069 have indeed been shown to retain their potency on the zebrafish receptor.

For both antagonist and agonists, the differences observed between the human and zebrafish receptors were recapitulated when testing on the human receptor bearing the zebrafish pocket mutations (Table 1). This result indicates that the reduction in potency is related to the pocket differences between human and zebrafish and not to any other aspect of the zebrafish receptor.

The affinity hikes observed for the different ligands between the human and zebrafish receptor underscore the importance of validating the functionality of ligands on the receptor of interest prior to setting up an animal model.

The selected OXR antagonists and agonists were then assessed in high-throughput behavioral experiments using zebrafish larvae, since sleep in zebrafish is characterized primarily by behavioral criteria. Two behavioral assays were developed: a night assay, since the main sleep phase of zebrafish larvae is during the night, and a dark-light assay, since lower responsiveness to external stimuli (i.e., dark-light transitions) is associated with sleep behavior in zebrafish larvae.

Overall, the data show that a high-throughput assay that records the locomotor activity of the animals during 14 h throughout the evening, night and morning was able to distinguish between OXR agonists and OXR antagonists active on the zebrafish OX2R (Figures 4, 5). All three antagonists reduced zebrafish locomotor activity during the Night 1 period, and two out of three reduced their activity in the Morning phase. Two out of three agonists increased zebrafish locomotor activity, especially during the Night 2 period. The apparent inactivity of TAK-925 in zebrafish can be explained by the reduced potency relative to the human receptor, related to receptor pocket differences, as demonstrated in the IP-One functional test. These differences must be kept in mind when using zebrafish OXR or zebrafish behavioral assays in screening for OXR (ant)agonists as novel hypnotics and psychostimulants for humans. Moreover, the difference in *vivo* activity between C15454 and C19069, in spite of their comparable *in vitro* potency, indicates that *in vitro* potency is not a sufficient condition to observe activity in a zebrafish animal model, and that parameters such as MTC, solubility and passive permeability should be taken into account as well. Interestingly, metabolic instability should not be an issue as the compounds are administered *via* immersion, thus enabling the evaluation of *in vivo* activity of compounds that are labile in rodent animal models.

Surprisingly, a locomotor assay with alternating 30 min dark-light transitions throughout the day was unable to distinguish between OXR agonists and antagonists active on the zebrafish OX2R (Figures 6, 7). Both antagonists and agonists, except TAK-925, lowered the behavioral response of zebrafish larvae after a light to dark transition, especially in the Dark 1 phase. This lower responsiveness to external stimuli (i.e., light to dark transitions) has been associated with sleep behavior in zebrafish, and thus would be also expected for OXR antagonists, which promote sleep in humans (Levitas-Djerbi and Appelbaum, 2017).

In case of OXR agonists, the unexpected inhibitory effect can be explained by competition for OXR binding of OXR agonists with endogenous orexins, the natural ligands of OXR, which is most likely to be present at higher levels during the day. The difference between the observed inhibitory effect during the day compared to the excitatory effect on zebrafish behavior of OX2R agonists during the evening and the night therefore implies that the circadian rhythm influences the effects of OX2R agonists on zebrafish behavior. This is possibly due to differences in synaptic rearrangement or orexin expression and release during night and day (Azeez et al., 2018; Ventzke et al., 2019).

## 5 Conclusion

We demonstrated that the functional IP-One test is a good predictor of biological activity *in vivo* through a test of small molecule OXR agonists and antagonists on human and zebrafish OX2Rs, and behavioral assays using zebrafish larvae. The behavioral data show that a high-throughput assay that records the locomotor activity of zebrafish during 24 h, throughout the evening, night and morning, is able to distinguish between OXR agonists and OXR antagonists active on the zebrafish OXR. Conversely, a locomotor assay with alternating 30 min dark-light transitions throughout the day is not able to distinguish these compounds, implying the importance of the circadian rhythm.

Overall, the results demonstrate that a functional IP-one test in combination with a behavioral assay using zebrafish is well-suited as a discovery platform to find novel compounds that target OXRs for the treatment of sleep disorders, including insomnia and narcolepsy.

## Data Availability

The original contributions presented in the study are included in the article/Supplementary Material, further inquiries can be directed to the corresponding authors.

## References

[B1] Adamantidis A. R. Schmidt M. H. Carter M. E. Burdakov D. Peyron C. Scammell T. E. (2020). A circuit perspective on narcolepsy. Sleep 43, zsz296. 10.1093/sleep/zsz296 31919524 PMC7215265

[B2] Ahmad S. F. Buckley A. W. Glaze D. G. (2021). Neurology of sleep. Neurol. Clin. 39, 867–882. 10.1016/j.ncl.2021.04.007 34215391

[B3] Azeez I. A. Del Gallo F. Cristino L. Bentivoglio M. (2018). Daily fluctuation of orexin neuron activity and wiring: The challenge of "chronoconnectivity. Front. Pharmacol. 9, 1061. 10.3389/fphar.2018.01061 30319410 PMC6167434

[B4] Barateau L. Dauvilliers Y. (2019). Recent advances in treatment for narcolepsy. Ther. Adv. Neurol. Disord. 12, 1756286419875622. 10.1177/1756286419875622 31632459 PMC6767718

[B5] Barker E. C. Flygare J. Paruthi S. Sharkey K. M. (2020). Living with narcolepsy: Current management strategies, future prospects, and overlooked real-life concerns. Nat. Sci. Sleep. 12, 453–466. 10.2147/NSS.S162762 32765142 PMC7371435

[B6] Bassetti C. L. A. Adamantidis A. Burdakov D. Han F. Gay S. Kallweit U. (2019). Narcolepsy - clinical spectrum, aetiopathophysiology, diagnosis and treatment. Nat. Rev. Neurol. 15, 519–539. 10.1038/s41582-019-0226-9 31324898

[B7] Bennett T. Bray D. Neville M. W. (2014). Suvorexant, a dual orexin receptor antagonist for the management of insomnia. P T. 39, 264–266. 24757363 PMC3989084

[B8] Bruni G. Lakhani P. Kokel D. (2014). Discovering novel neuroactive drugs through high-throughput behavior-based chemical screening in the zebrafish. Front. Pharmacol. 5, 153. 10.3389/fphar.2014.00153 25104936 PMC4109429

[B9] Chemelli R. M. Willie J. T. Sinton C. M. Elmquist J. K. Scammell T. Lee C. (1999). Narcolepsy in orexin knockout mice: Molecular genetics of sleep regulation. Cell 98, 437–451. 10.1016/s0092-8674(00)81973-x 10481909

[B10] Copmans D. Kildgaard S. Rasmussen S. A. Ślęzak M. Dirkx N. Partoens M. (2019). Zebrafish-based discovery of antiseizure compounds from the north sea: Isoquinoline alkaloids TMC-120a and TMC-120B. Mar. Drugs 17, 607. 10.3390/md17110607 PMC6891649 31731399

[B11] Copmans D. Orellana-Paucar A. M. Steurs G. Zhang Y. Ny A. Foubert K. (2018). Methylated flavonoids as anti-seizure agents: Naringenin 4', 7-dimethyl ether attenuates epileptic seizures in zebrafish and mouse models. Neurochem. Int. 112, 124–133. 10.1016/j.neuint.2017.11.011 29174382

[B12] Cox C. D. Breslin M. J. Whitman D. B. Schreier J. D. Mcgaughey G. B. Bogusky M. J. (2010). Discovery of the dual orexin receptor antagonist [(7R)-4-(5-chloro-1, 3-benzoxazol-2-yl)-7-methyl-1, 4-diazepan-1-yl] [5-methyl-2-(2H-1, 2, 3-triazol-2-yl)phenyl]methanone (MK-4305) for the treatment of insomnia. J. Med. Chem. 53, 5320–5332. 10.1021/jm100541c 20565075

[B13] Dye T. J. Gurbani N. Simakajornboon N. (2018). Epidemiology and pathophysiology of childhood narcolepsy. Paediatr. Respir. Rev. 25, 14–18. 10.1016/j.prrv.2016.12.005 28108192

[B14] Elbaz I. Foulkes N. S. Gothilf Y. Appelbaum L. (2013). Circadian clocks, rhythmic synaptic plasticity and the sleep-wake cycle in zebrafish. Front. Neural Circuits 7, 9. 10.3389/fncir.2013.00009 23378829 PMC3561628

[B15] Elbaz I. Levitas-Djerbi T. Appelbaum L. (2017). The hypocretin/orexin neuronal networks in zebrafish. Curr. Top. Behav. Neurosci. 33, 75–92. 10.1007/7854_2016_59 28012092

[B16] Hong C. Byrne N. J. Zamlynny B. Tummala S. Xiao L. Shipman J. M. (2021). Structures of active-state orexin receptor 2 rationalize peptide and small-molecule agonist recognition and receptor activation. Nat. Commun. 12, 815. 10.1038/s41467-021-21087-6 33547286 PMC7864924

[B17] Irukayama-Tomobe Y. Ogawa Y. Tominaga H. Ishikawa Y. Hosokawa N. Ambai S. (2017). Nonpeptide orexin type-2 receptor agonist ameliorates narcolepsy-cataplexy symptoms in mouse models. Proc. Natl. Acad. Sci. U. S. A. 114, 5731–5736. 10.1073/pnas.1700499114 28507129 PMC5465922

[B18] Isberg V. De Graaf C. Bortolato A. Cherezov V. Katritch V. Marshall F. H. (2015). Generic GPCR residue numbers - aligning topology maps while minding the gaps. Trends Pharmacol. Sci. 36, 22–31. 10.1016/j.tips.2014.11.001 25541108 PMC4408928

[B19] Kaushik M. K. Aritake K. Cherasse Y. Imanishi A. Kanbayashi T. Urade Y. (2021). Induction of narcolepsy-like symptoms by orexin receptor antagonists in mice. Sleep 44, zsab043. 10.1093/sleep/zsab043 33609365

[B20] Lazareno S. Birdsall N. J. (1993). Estimation of competitive antagonist affinity from functional inhibition curves using the Gaddum, Schild and Cheng-Prusoff equations. Br. J. Pharmacol. 109, 1110–1119. 10.1111/j.1476-5381.1993.tb13737.x 8401922 PMC2175764

[B21] Leung L. C. Wang G. X. Madelaine R. Skariah G. Kawakami K. Deisseroth K. (2019). Neural signatures of sleep in zebrafish. Nature 571, 198–204. 10.1038/s41586-019-1336-7 31292557 PMC7081717

[B22] Levitas-Djerbi T. Appelbaum L. (2017). Modeling sleep and neuropsychiatric disorders in zebrafish. Curr. Opin. Neurobiol. 44, 89–93. 10.1016/j.conb.2017.02.017 28414966

[B23] Li S. B. de Lecea L. (2020). The hypocretin (orexin) system: From a neural circuitry perspective. Neuropharmacology 167, 107993. 10.1016/j.neuropharm.2020.107993 32135427

[B24] Lin L. Faraco J. Li R. Kadotani H. Rogers W. Lin X. (1999). The sleep disorder canine narcolepsy is caused by a mutation in the hypocretin (orexin) receptor 2 gene. Cell 98, 365–376. 10.1016/s0092-8674(00)81965-0 10458611

[B25] Mahoney C. E. Cogswell A. Koralnik I. J. Scammell T. E. (2019). The neurobiological basis of narcolepsy. Nat. Rev. Neurosci. 20, 83–93. 10.1038/s41583-018-0097-x 30546103 PMC6492289

[B26] Mezeiova E. Janockova J. Konecny J. Kobrlova T. Benkova M. Dolezal R. (2020). From orexin receptor agonist YNT-185 to novel antagonists with drug-like properties for the treatment of insomnia. Bioorg. Chem. 103, 104179. 10.1016/j.bioorg.2020.104179 32891860

[B27] Mieda M. (2017). The roles of orexins in sleep/wake regulation. Neurosci. Res. 118, 56–65. 10.1016/j.neures.2017.03.015 28526554

[B28] Miyawaki I. (2020). Application of zebrafish to safety evaluation in drug discovery. J. Toxicol. Pathol. 33, 197–210. 10.1293/tox.2020-0021 33239838 PMC7677624

[B29] Nishimura Y. Okabe S. Sasagawa S. Murakami S. Ashikawa Y. Yuge M. (2015). Pharmacological profiling of zebrafish behavior using chemical and genetic classification of sleep-wake modifiers. Front. Pharmacol. 6, 257. 10.3389/fphar.2015.00257 26578964 PMC4630575

[B30] Nishino S. Ripley B. Overeem S. Lammers G. J. Mignot E. (2000). Hypocretin (orexin) deficiency in human narcolepsy. Lancet 355, 39–40. 10.1016/S0140-6736(99)05582-8 10615891

[B31] Prober D. A. Rihel J. Onah A. A. Sung R. J. Schier A. F. (2006). Hypocretin/orexin overexpression induces an insomnia-like phenotype in zebrafish. J. Neurosci. 26, 13400–13410. 10.1523/JNEUROSCI.4332-06.2006 17182791 PMC6675014

[B32] Ramar K. Olson E. J. (2013). Management of common sleep disorders. Am. Fam. Physician 88, 231–238. 23944726

[B33] Sun Y. Tisdale R. K. Kilduff T. S. (2021). Hypocretin/orexin receptor pharmacology and sleep phases. Front. Neurol. Neurosci. 45, 22–37. 10.1159/000514963 34052813 PMC8171809

[B34] Tisdale R. K. Yamanaka A. Kilduff T. S. (2021). Animal models of narcolepsy and the hypocretin/orexin system: Past, present, and future. Sleep 44, zsaa278. 10.1093/sleep/zsaa278 33313880 PMC8193560

[B35] Toth L. A. Bhargava P. (2013). Animal models of sleep disorders. Comp. Med. 63, 91–104. 23582416 PMC3625050

[B36] Ventzke K. Oster H. Jöhren O. (2019). Diurnal regulation of the orexin/hypocretin system in mice. Neuroscience 421, 59–68. 10.1016/j.neuroscience.2019.10.002 31678347

[B37] Yukitake H. Fujimoto T. Ishikawa T. Suzuki A. Shimizu Y. Rikimaru K. (2019). TAK-925, an orexin 2 receptor-selective agonist, shows robust wake-promoting effects in mice. Pharmacol. Biochem. Behav. 187, 172794. 10.1016/j.pbb.2019.172794 31654653

[B38] Zeitzer J. M. (2021). The neurobiological underpinning of the circadian wake signal. Biochem. Pharmacol. 191, 114386. 10.1016/j.bcp.2020.114386 33359009

[B39] Zhdanova I. V. (2006). Sleep in zebrafish. Zebrafish 3, 215–226. 10.1089/zeb.2006.3.215 18248262

