# Supplementary material for: Modulation of sleep behavior in zebrafish larvae by pharmacological targeting of the orexin receptor
Source: Front Pharmacol. 2022 Oct 10;13:1012622. doi: 10.3389/fphar.2022.1012622 (PMC9632972; doi:10.3389/fphar.2022.1012622)
Supplement: Supplementary file 1 [file Table1.pdf]

Supplementary Material

1 Supplementary Data

1.1 Supplementary Tables

OXR antagonists	MTC (µM) Dark-Light assay	MTC (µM) Night assay	Max. solubility (µM)
Suvorexant	5	3	50
TCS-1102	30	15	>100
SB-674042	25	12.5	>100
**OXR agonists**	**MTC (µM) Dark-Light assay**	**MTC (µM) Night assay**	**Max. solubility (µM)**
C15454	150	100	500
C19069	100	50	>500
TAK-925	25	25	50

Supplementary Table 1. The maximum tolerated concentration (MTC) of orexin receptor (OXR) antagonists and agonists in 6 dpf zebrafish larvae.
